# Maternal methyl-donor supplementation and hypothalamic methylation stability

**DOI:** 10.1530/EC-25-0790

**Published:** 2026-07-15

**Authors:** Thomas Gerhard Brune, Bettina Schloesser, Marianne Volleth, Martin Zenker

**Affiliations:** ^1^ University Hospital Magdeburg, Department of Pediatrics, Magdeburg, Saxony-Anhalt, Germany; ^2^ Klinikum Lippe Detmold, Department of Pediatrics, Detmold, North Rhine-Westphalia, Germany; ^3^ University Hospital Magdeburg, Institute for Human Genetics, Magdeburg, Saxony-Anhalt, Germany

**Keywords:** DNA methylation, epigenetic programming, one-carbon metabolism, hypothalamus, nutrition

## Abstract

**Background:**

Maternal nutrition can shape fetal epigenetic programming with long-term health effects. This study tested whether central and peripheral tissues differ in responsiveness to maternal methyl-donor supplementation.

**Methods:**

C57BL/6 dams received either standard chow (control) or a methyl-donor-enriched diet during pregnancy. Offspring were allocated to three groups: i) control (mother and offspring on standard chow), ii) prenatal exposure (mother on methyl-donor diet during pregnancy, offspring on standard chow thereafter), and iii) combined exposure (mother on methyl-donor diet during pregnancy and lactation, offspring continued methyl-donor diet postnatally). Global DNA methylation was measured by ELISA in the brain and hypothalamus (*n* = 10) and in the liver, spleen, and heart (*n* = 5). Exploratory hypothalamic gene expression analysis compared the prenatal exposure group vs controls (*n* = 4).

**Results:**

Prenatal supplementation was associated with increased global 5-methylcytosine levels in the brain and hypothalamus (both *P* < 0.01). In the hypothalamus, this elevation persisted irrespective of later diet, whereas peripheral organs displayed ongoing plasticity and responded strongly to postnatal exposure. Microarray analysis identified 36 differentially expressed hypothalamic transcripts, including genes previously linked to methylation-related pathways.

**Conclusion:**

Maternal methyl-donor supplementation is associated with a stable hypothalamic methylation profile established during intrauterine life, while peripheral organs remain epigenetically adaptable. These findings reveal an organ-specific divergence in developmental programming, with exploratory gene expression suggesting functional consequences.

## Introduction

Early-life nutrition exerts a profound influence on lifelong health, a concept now widely known as the developmental origins of health and disease (DOHaD) (1, 2, 3). Epigenetic mechanisms – including DNA methylation and chromatin remodeling – provide a molecular basis by which environmental and nutritional factors shape gene expression patterns and physiological set-points across development (4, 5, 6). Nutrients of the one-carbon (1-C) metabolism (folate, choline, betaine, vitamin B12, and methionine) supply methyl groups and cofactors for methyltransferases and thereby directly influence the genomic methylation landscape (7, 8, 9, 10, 11, 12). In humans, periconceptional folate status and related 1-C nutrients have been linked to persistent epigenetic marks and metabolic phenotypes in offspring (13, 14, 15).

Most studies to date have focused on peripheral metabolic organs – particularly liver, adipose tissue, and blood – where postnatal diet has been shown to influence locus-specific DNA methylation and gene expression, for example, in pathways of lipid and glucose metabolism and adipogenesis (16, 17). These findings indicate that peripheral tissues remain epigenetically plastic in response to dietary exposures throughout life. In contrast, much less is known about the hypothalamus, the central regulator of energy balance and endocrine integration, despite substantial evidence that early hormonal and nutritional cues shape hypothalamic development and long-term metabolic control (18, 19, 20, 21). Recent reviews of 1-C metabolism also highlight the need to consider brain region specificity and developmental timing when interpreting nutritional programming effects (8, 9, 17, 20).

Against this background, we investigated whether maternal methyl-donor supplementation during pregnancy induces organ- and timing-specific epigenetic programming in the offspring. Specifically, we quantified global DNA methylation across central (brain and hypothalamus) and peripheral tissues (e.g. liver and spleen) and asked whether intrauterine exposure establishes patterns that persist or adapt to the postnatal dietary environment. In addition, we profiled hypothalamic gene expression to determine whether global methylation shifts translate into coherent transcriptional changes, benchmarking selected findings against published hypothalamic datasets for robustness (22).

Based on developmental neurobiology, we reasoned that the hypothalamus may establish a relatively stable epigenetic profile during intrauterine life. The hypothalamus is known to undergo critical developmental programming in response to early hormonal and nutritional cues (18, 19, 20, 21) and has been described as a lifelong regulator of metabolic set-points (19). In contrast, peripheral organs, such as liver and adipose tissue, remain metabolically and epigenetically plastic throughout life (16, 17). We therefore hypothesized that maternal methyl-donor supplementation would induce a persistent hypothalamic methylation set-point, whereas peripheral tissues would continue to show postnatal adaptability.

## Materials and methods

### Animals

C57BL/6 wild-type mice (origin: Jackson Laboratory; bred in-house at the University of Magdeburg) were housed under standardized conditions (inverse light cycle, 22–24°C, access to food and water *ad libitum*). All procedures complied with Directive 2010/63/EU and were approved by the local authorities in Halle (Saale). Six breeding pairs were set up at six months of age.

### Diet composition and sourcing

Diets were manufactured by ssniff® (Soest, Germany). The standard diet was ssniff® R/M-H, a maintenance diet for mice. The base chow composition (ssniff® R/M-H) was as follows:

Energy: gross energy 16.3 MJ/kg and metabolizable energy 12.8 MJ/kg; macronutrient distribution: ∼58% carbohydrate, 33% protein, and 9% fat.

Selected one-carbon-related components (per kg): folic acid 7 mg; vitamin B12 100 μg; vitamin B6 21 mg; methionine 0.30% w/w (∼3 g/kg); choline chloride 2,990 mg.

The methyl-donor-supplemented chow was formulated to achieve approximately fourfold higher levels of these components (per kg diet, targets): folic acid ∼28 mg; vitamin B12 ∼400 μg; vitamin B6 ∼84 mg; methionine ∼1.2% w/w (∼12 g/kg); choline chloride ∼12,000 mg, while maintaining identical energy density and macronutrient composition. The methyl-donor-enriched diet was designed to target one-carbon metabolism as an integrated system. The supplemented nutrients act as interdependent cofactors within the methylation cycle and collectively determine methyl-group availability. Accordingly, combined supplementation was chosen to modulate overall methylation capacity rather than to investigate isolated effects of individual components. The diet remained isocaloric and isonitrogenous relative to the base chow. Macronutrient distribution (protein, fat, and carbohydrate), fiber, minerals, and non-target vitamins were matched to the control diet.

The six breeding pairs were allocated to three exposure paradigms:

- control – dams and offspring received standard chow during gestation, lactation, and postnatal life;
- gestation-only methyl-donor supplementation – dams received standard chow supplemented with methyl donors during gestation only; thereafter, dams and offspring received standard chow;
- gestation plus offspring supplementation – dams received methyl-donor-supplemented chow during gestation and lactation, and offspring continued methyl-donor-supplemented chow after birth and after weaning until the experimental endpoint (adolescence/3 months).

### Sample collection

Only female offspring were included in global DNA methylation analyses. F1 offspring were sacrificed at three months of age by CO_2_ inhalation. Organs (heart, spleen, liver, and brain) were excised, snap-frozen, and stored in liquid nitrogen (−196°C). The hypothalamus was micro-dissected using standardized anatomical landmarks (optic chiasm and mammillary bodies as rostral/caudal borders). Per group, *n* = 5 samples were analyzed for heart, spleen, and liver and *n* = 10 for brain and hypothalamus.

### Global DNA methylation

Genomic DNA was extracted from organ tissue using the NucleoBond® kit (Macherey-Nagel, Germany). Global 5-methylcytosine content was quantified with the Methylamp™ Global DNA Methylation Quantification ELISA kit (EpigenTek, USA) according to the manufacturer’s protocol. This assay immobilizes genomic DNA on assay wells and detects methylated cytosines using a capture/detection antibody system with colorimetric readout at 450 nm. Equal amounts of genomic DNA (200 ng per well) were used for all samples following prior quantification and normalization of DNA concentrations.

Results are expressed relative to the internal kit standard, which is set to 100%. Because this standard represents an assay-specific positive control rather than an absolute biological maximum, values > 100% are technically possible. Importantly, the ELISA has been validated against HPLC–MS according to the manufacturer. For each sample, measurements were performed in technical triplicates, and the median of the three replicate values was used for statistical analysis.

### Hypothalamus RNA isolation and microarray analysis

Hypothalamic tissue was micro-dissected from frozen brains. Total RNA was isolated using QIAzol and RNeasy columns (Qiagen, Germany), including on-column DNase digestion. RNA quality was assessed by NanoDrop spectrophotometry and BioAnalyzer 2100 (Agilent, USA); only samples with RNA integrity number (RIN) ≥ 7.0 were included. Of six hypothalamic samples initially prepared, four passed all QC criteria and entered expression analysis (two offspring from methyl-donor-supplemented dams (female) and two offspring from control dams (male)).

The inclusion of samples from both sexes was deliberately used as an internal validation strategy, enabling comparison with the large sex-difference dataset of Mozhui *et al.* (22), generated on the same microarray platform (see the section titled Results). The aim of this comparison was to assess whether our dataset reproduces established sex-dimorphic expression patterns, thereby providing an internal quality control despite the small sample size. Importantly, sex was not analyzed as a biological variable, and the study design does not allow separation of sex and dietary effects. Therefore, gene expression findings are interpreted solely at an exploratory level and not as evidence for causal dietary regulation.

From 200 ng total RNA, cRNA was generated using the Low-RNA Input Linear Amplification kit (Agilent) and hybridized to the Agilent Mouse Gene Expression v2 4 × 44K Microarray (G4846A). Arrays were scanned with an Agilent DNA Microarray Scanner (G2565A), and raw data were extracted with Feature Extraction Software v10.5. Data preprocessing included background correction, inter-array scaling, and median centering per gene. Genes with ≥threefold differential expression and *P* < 0.05 were considered differentially expressed. This threshold was chosen as a stringent filter to identify robust exploratory candidates despite the small sample size.

### Statistics

Data from global DNA methylation assays are presented as mean ± SD. The normality of distributions was assessed using the Shapiro–Wilk test. For normally distributed data, unpaired two-tailed Student’s *t*-tests were applied (GraphPad Prism®, USA). A *P*-value <0.05 was considered statistically significant.

For hypothalamic expression analysis, four samples (two methyl-donor vs two control) entered the microarray comparison. Data preprocessing included background correction using Agilent Feature Extraction software, followed by inter-array normalization and median centering. Signal intensities were normalized using the default scaling procedures provided by the Agilent platform. Expression data were processed with Agilent Feature Extraction software, which calculates fold changes and nominal *P*-values from probe-level replicates. For comparison with the dataset of Mozhui *et al.* (Table 1), fold changes were expressed as log2-transformed values (log2FC) to ensure methodological comparability with the published data. Fold changes for the primary analysis (Table 2) are reported as linear fold differences relative to controls and were calculated from normalized signal intensities. Differentially expressed transcripts were defined by a threshold of ≥threefold change with *P* < 0.05 relative to controls.

**Table 1:** Transcripts with differential expression between males and females in the hypothalamus comparison of Mozhui *et al.* (22) (*n* = 78) and the present study (*n* = 4). Fold changes are expressed as log2-transformed values (log2FC) for both datasets to allow direct comparison. Positive values indicate higher expression in females; negative values indicate higher expression in males.

Gene	Description	Chromosome	Mozhui *et al.* (22)		The present study	
			*P*-value	log2FC	*P*-value	log2FC
Xist	Inactive X-specific transcript	X	<0.01	41.82	<0.01	8.27
Kdm6a	Histone demethylase (UTX)	X	<0.01	1.36	<0.05	0.87
Eif2s3x	Translation initiation factor	X	<0.01	1.31	<0.05	−2.07
Ddx3x	RNA helicase	X	<0.01	1.27	<0.01	0.77
Kdm5c	Histone demethylase	X	<0.01	1.27	<0.05	0.29
2610029G23Rik	Novel X-inactivation escapee	X	<0.01	1.16	n.s.*	—
Tac2	Tachykinin peptide hormone	10	<0.01	1.32	n.s.	—
Esr1	Estrogen receptor	10	<0.01	1.29	n.s.	—
Gm9884	Predicted gene	1	<0.01	1.21	n.s.	—
Gpr88	G protein-coupled receptor 88	3	<0.01	1.19	n.s.	—
Socs2	Suppressor of cytokine signaling 2	12	<0.01	1.18	n.s.	—
Vmn1r90	Vomeronasal receptor	7	<0.01	1.18	n.s.	—
Npbwr1	Neuropeptide receptor	1	<0.01	1.16	n.s.	—
Oxtr	Oxytocin receptor	6	<0.01	1.14	n.s.	—
Nmur2	Neuromedin peptide receptor	11	<0.01	1.12	n.s.	—
Nmbr	Neuromedin receptor	10	<0.01	1.12	n.s.	—
Gpr83	G protein-coupled receptor	9	<0.01	1.12	n.s.	—
Cish	Cytokine-inducible SH2 protein	9	<0.01	1.11	n.s.	—
Stra6	Stimulated by retinoic acid gene 6	9	<0.01	1.10	n.s.	—
Eif2s3y	Translation initiation factor	Y	<0.01	−17.25	<0.01	−6.97
Ddx3y	RNA helicase	Y	<0.01	−17.22	<0.01	−7.65
Uty	Histone demethylase	Y	<0.01	−13.34	<0.01	−1.49
Kdm5d	Histone demethylase	Y	<0.01	−6.23	<0.01	−3.47
Rbm3	RNA-binding motif gene	X	<0.01	−1.17	<0.01	−1.10
Arhgap36	Rho GTPase-activating protein	X	<0.01	−1.16	n.s.	—
Sytl4	Synaptotagmin-like gene	X	<0.01	−1.13	n.s.	—
Lamp5	Lysosomal glycoprotein	2	<0.01	−1.23	n.s.	—
Serpina3n	Serine protease inhibitor	12	<0.01	−1.11	n.s.	—
Adamts2	Procollagen metallopeptidase	11	<0.01	−1.10	n.s.	—

* Fold change values are shown as log2 fold change.

**Table 2:** Differentially expressed hypothalamic genes in offspring exposed to a methyl-rich maternal diet (AbsFoldChange ≥ 3, *P* < 0.05). Genes are grouped according to functional clusters adapted from Mozhui *et al.* (22).

Gene symbol	Description	Locus	Fold change
**Endocrine/nutrient axis**			
Folr1	Folate receptor 1	Chr 7	+3.8
Nat3	N-acetyltransferase 3	Chr 8	+4.3
Slc22a1	Solute carrier family 22 member 1	Chr 17	−3.1
Ttr	Transthyretin	Chr 18	+7.6
**Growth/transcriptional cluster**			
Arhgef2	Rho guanine nucleotide exchange factor 2	Chr 4	−3.3
Ccdc85a	Coiled-coil domain-containing 85A	Chr 6	−3.0
Dmrta1	Doublesex- and mab-3-related transcription factor-like A1	Chr 4	−9.4
Fignl1	Fidgetin-like 1	Chr 11	−6.1
Gzmk	Granzyme K	Chr 13	−3.6
Hist1h2bm	Histone cluster 1 H2bm	Chr 13	−3.3
Hmgn5	High mobility group nucleosome-binding domain 5	Chr X	−3.2
Itch	Itchy, E3 ubiquitin protein ligase	Chr 2	−3.1
Klhl9	Kelch-like family member 9	Chr 3	−3.4
Lmo4	LIM domain only 4	Chr 3	−3.0
Nfkb1	Nuclear factor of kappa light polypeptide gene enhancer in B-cells	Chr 3	−3.0
Plekhg3	Pleckstrin homology domain-containing family G member 3	Chr 12	−3.2
Ptbp1	Polypyrimidine tract-binding protein 1	Chr 10	−3.2
Ptprv	Protein tyrosine phosphatase, receptor type V	Chr 1	+3.1
Rrn3	RRN3 RNA polymerase I transcription factor	Chr 16	−3.1
Smarca2	SWI/SNF-related, matrix-associated, actin-dependent regulator of chromatin, subfamily a, member 2	Chr 9	−3.0
Sox4	SRY-box transcription factor 4	Chr 13	−3.1
Usp9x	Ubiquitin-specific peptidase 9, X-linked	Chr X	−3.0
Zfp36l1	Zinc finger protein 36, C3H type-like 1	Chr 4	−3.0
Zfp689	Zinc finger protein 689	Chr 7	−3.4
**Structural/peptidergic cluster**			
Adhfe1	Alcohol dehydrogenase, iron containing 1	Chr 3	−3.2
Atp6v1g2	ATPase, H+ transporting, V1 subunit G2	Chr 7	−3.1
Ctsz	Cathepsin Z	Chr 2	−3.5
Ecrg4	Augurin (1500015O10Rik)	Chr 1	+6.2
Kif20b	Kinesin family member 20B	Chr 19	+3.3
Pkhd1l1	Polycystic kidney and hepatic disease 1-like 1	Chr 15	+3.0
Ripk4	Receptor-interacting serine–threonine kinase 4	Chr 16	−3.6
Serpina11	Serine (or cysteine) peptidase inhibitor 11	Chr 12	+7.3
Tmprss11e	Transmembrane protease, serine 11e	Chr 5	−4.4
Tubb2a	Tubulin beta 2A	Chr 13	−3.0
**Genes without established function**			
A130019P10Rik	RIKEN cDNA	Chr 10	−3.4
Gm428	Predicted gene 428	Chr 4	−3.1
Olfr1256	Olfactory receptor 1256	Chr 2	−3.7

Sex-chromosomal control genes described by Mozhui *et al.* (e.g. Xist, Ddx3y, Eif2s3y, and Kdm5d) were excluded from this table, as sex and diet were fully confounded in our design. Only Eif2s3x, showing opposite regulation compared with Mozhui *et al.*, is retained for discussion. Positive fold change values (+) indicate upregulation in the diet group compared with controls, and negative values (−) indicate downregulation.

## Results

### Global DNA methylation

**Figure 1:**
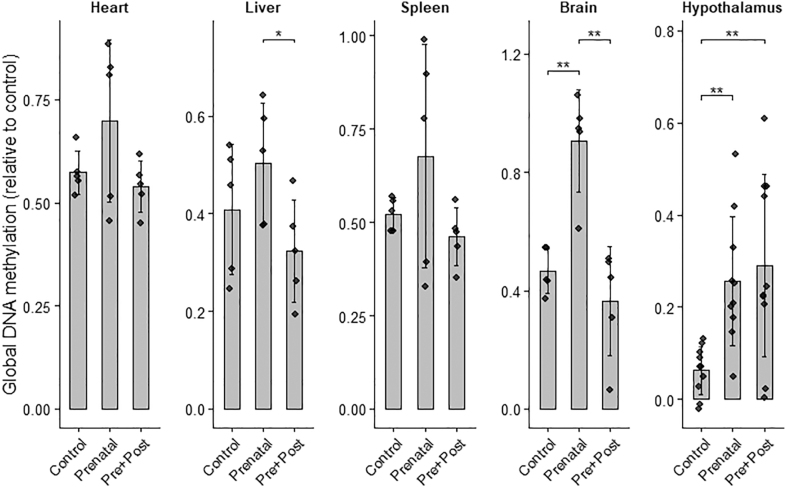
Global DNA methylation in different tissues of offspring at 3 months of age following maternal methyl-donor supplementation during distinct exposure windows (control, prenatal, and pre + postnatal supplementation). Global 5-methylcytosine (5mC) levels were quantified using an ELISA-based assay and are expressed relative to the internal assay standard. Data are presented as mean ± SD, with individual data points representing biological replicates. Brain and hypothalamus, *n* = 10 per group; heart, liver, and spleen, *n* = 5 per group. Statistical significance was assessed using unpaired two-tailed Student’s *t*-tests; significant differences are indicated in the figure (*P < 0.05, **P < 0.01).

Global DNA methylation differed across tissues and depended on the timing of maternal methyl-supplemented diet (Fig. 1). Baseline global DNA methylation levels (controls) in the hypothalamus were significantly lower compared with all other tissues (adjusted *P* < 0.01 for all comparisons). Differences among the remaining tissues were less consistent and not the primary focus of the present analysis. In the brain, prenatal supplementation resulted in markedly higher methylation compared with both control (*P* < 0.01) and pre + postnatal exposure (*P* < 0.01), highlighting a high degree of postnatal plasticity. In contrast, the hypothalamus showed increased methylation only after prenatal supplementation compared with control (*P* < 0.01), but no further changes postnatally, indicating a relative stability of global methylation levels compared with controls, as measured by ELISA. Since this assay provides relative rather than absolute 5mC content, our conclusions refer to comparative differences within tissues across exposure conditions rather than absolute quantitative set-points or direct comparisons between tissues. In the liver, prenatal supplementation led to higher methylation compared with postnatal exposure (*P* < 0.05), whereas differences between control and prenatal or postnatal exposure were less pronounced. Heart and spleen displayed intermediate patterns with moderate increases after prenatal supplementation but without significant differences among groups.

### Quality control of the expression analysis

To assess the reliability of the hypothalamic array data, we used sex-specific transcripts as an internal quality control. In line with the large reference dataset by Mozhui *et al.* (22), our profiles (*n* = 4) reproduced the same male–female differences in hypothalamic gene expression (Table 1). To avoid confounding between diet and sex, we *a priori* excluded five sex-chromosomal control transcripts (Xist, Ddx3y, Eif2s3y, Kdm5d, and Rbm3) from further interpretation. Several autosomal sex-biased genes reported by Mozhui (e.g. Tac2, Esr1, Gm9884, and Lamp5) were also detected in our dataset with concordant trends but did not meet our differential expression threshold of ≥threefold and were therefore not included in Table 2. The only exception was Eif2s3x, which showed opposite regulation compared with Mozhui *et al.* (22) and was therefore retained for discussion.

### Hypothalamic gene expression in offspring of mothers with methyl-donor-enriched diet

The results of the expression analysis are summarized in Table 1. After exclusion of five sex-specific transcripts (see the section titled Methods), a total of 36 genes were differentially expressed by at least threefold in the diet group compared with controls. Positive fold change values (+) indicate upregulation in the diet group relative to controls, whereas negative values (−) indicate downregulation.

Functional grouping revealed four major clusters:

- Endocrine/nutrient axis: several genes involved in hormone and nutrient handling were altered. Ttr showed the strongest upregulation. Folr1, Srd5a2, and Nat3 were also increased, whereas Slc22a1 was decreased, suggesting modulation of hormone transport and steroid metabolism.
- Growth/transcriptional cluster: genes related to transcriptional and growth regulation were affected. Braf and Med12l were upregulated, whereas Rrn3 (TIF-IA), Ptbp1, Nfkb1, and Plekhg3 were downregulated. Additional changes included Zfp689, Itch, and Ptprv, pointing to altered transcriptional control.
- Structural/peptidergic cluster: genes associated with cellular structure and peptide signaling were also altered. Kif20b, Pkhd1l1, and Cmya5 were upregulated, while Ripk4 was downregulated. Ecrg4 and Serpina11 were increased, whereas Tmprss11e was decreased, indicating changes in structural integrity and neuropeptide-related pathways.
- Genes without established function: several transcripts without clear annotation were altered, including 2010110K18Rik, 4930509J09Rik, A130019P10Rik, Gm428, and the olfactory receptor Olfr1256, highlighting the exploratory nature of the dataset.

## Discussion

Our observations are in line with previous work showing that early hormonal and nutritional signals durably shape hypothalamic development (18, 19, 20). A recent review by Kubant *et al.* (20) emphasized the need to consider hypothalamic epigenetic programming as a key mechanism within the DOHaD framework. A central finding of this study is the organ-specific divergence in global DNA methylation in response to maternal methyl-donor supplementation. While peripheral organs, such as liver and spleen, displayed dynamic changes that closely followed the postnatal diet, the hypothalamus maintained a persistently elevated methylation status once established during intrauterine life. Although the hypothalamus displayed lower baseline methylation levels compared with other tissues in our assay, this does not affect the interpretation, as analyses are based on relative within-tissue changes rather than absolute inter-tissue comparisons. This contrast between central stability and peripheral plasticity has, to our knowledge, not been previously reported and supports the hypothesis that the hypothalamus may function as an epigenetic set-point regulator, whereas peripheral tissues retain nutritional flexibility. This observation adds a new dimension to the DOHaD paradigm, which has traditionally emphasized peripheral metabolic organs as the primary sites of early-life nutritional programming (2, 3, 4).

Our finding of a stable hypothalamic methylation set-point is consistent with reports that early hormonal and nutritional cues durably shape hypothalamic circuits (18, 19, 20, 21). By contrast, the continued plasticity of peripheral tissues observed here agrees with previous evidence that liver and adipose epigenomes remain responsive to postnatal diet (16, 17). Similar organ-specific effects of methyl-donor supplementation have been described in liver and blood (7, 8, 9), whereas corresponding evidence for the hypothalamus has so far been limited.

A second key observation is the distinct effect of intrauterine versus postnatal exposure. Intrauterine supplementation imposed a stable hypothalamic methylation signature that persisted irrespective of the offspring’s later diet, whereas postnatal supplementation predominantly affected peripheral tissues. This timing- and organ-specific divergence underscores the concept of critical windows of developmental epigenetic plasticity (1, 11, 23). Our results therefore suggest that intrauterine one-carbon metabolism has qualitatively different consequences from postnatal supplementation, with permanent central programming but reversible peripheral adaptations.

Among the 36 differentially expressed hypothalamic transcripts, several downregulated genes have been reported to be repressed by locus-specific DNA methylation – namely SLC22A1 (promoter hypermethylation) (24), ADHFE1 (promoter hypermethylation) (25), and ZFP36L1 (enhancer hypermethylation) (26). This convergence supports the plausibility that maternal methyl-donor supplementation may influence hypothalamic gene expression through epigenetic regulation, although our assay does not resolve locus-specific methylation. The small sample size (*n* = 4) in the expression analysis inevitably limits statistical power. However, the use of a stringent fold change threshold (≥threefold, *P* < 0.05) and external benchmarking against the dataset of Mozhui *et al.* (22) supports the plausibility of the identified candidates. Still, the risk of false positives cannot be excluded, and findings should be regarded as exploratory. Independent validation of selected candidates using qPCR or locus-specific methylation assays will be required to confirm these findings in future studies.

By contrast, the upregulated transcripts are more likely to reflect indirect adaptations to the altered one-carbon metabolic milieu rather than direct methylation events. For example, increased Folr1 and Ttr expression may represent compensatory responses related to folate transport and central thyroid hormone handling, respectively. In humans, studies on maternal folate and child outcomes are heterogeneous, with both protective and adverse associations reported (23, 27), underscoring the complexity of translating these mechanisms. The marked upregulation of *Ttr*, encoding transthyretin, may link one-carbon metabolism to retinol signaling, a pathway implicated in adipocyte function and lipid homeostasis. Notably, increased retinoic acid receptor B gene methylation has been reported in Dunnigan-type lipodystrophy (28), underscoring the potential relevance of this axis for metabolic regulation.

As noted above, our results largely align with the sex-specific dataset of Mozhui *et al.* (22). One notable exception is Eif2s3x: whereas Mozhui *et al.* reported higher expression in the female hypothalamus, we observed a decrease under a methyl-rich diet. Given that Eif2s3x is an X-inactivation-escape gene in the brain, this discrepancy may reflect diet-induced alterations in escape/X-linked dosage control, compensatory mechanisms in translational initiation, or methodological factors and warrants further investigation.

Differentially expressed genes clustered into coherent functional groups – endocrine/nutrient handling, transcriptional and growth regulation, and structural/neuromodulatory pathways – consistent with key hypothalamic roles in energy balance and neuroendocrine control. Additional candidates, such as Nfkb1, Braf, and Slc22a1, suggest potential links to inflammatory signaling, growth pathways, and metabolite transport; however, these observations remain exploratory and require further validation. With respect to respiratory outcomes potentially linked to folate exposure, the evidence is heterogeneous, including a dose–response meta-analysis reporting increased risk of childhood asthma at higher maternal folic acid intake and cohort data with population-dependent effects (23, 27).

Together, our data reveal a striking dichotomy within the central nervous system: the whole brain remains epigenetically plastic and responsive to both intrauterine and postnatal methyl-donor exposure, whereas the hypothalamus establishes a stable methylation set-point during intrauterine life that resists later modulation. This model provides a mechanistic basis for how maternal diet may impose long-term hypothalamic ‘set-points’ that shape endocrine and metabolic regulation throughout life. Future work should include locus-specific methylation assays and sex-stratified, larger cohorts to validate these findings and define the functional consequences of maternal methyl-donor exposure.

## Declaration of interest

The authors declare that there is no conflict of interest that could be perceived as prejudicing the impartiality of the research reported.

## Funding

This research did not receive any specific grant from any funding agency in the public, commercial, or not-for-profit sector.
